# Functional midterm follow-up comparison of stemless total shoulder prostheses versus conventional stemmed anatomic shoulder prostheses using a 3D-motion-analysis

**DOI:** 10.1186/s12891-017-1835-3

**Published:** 2017-11-21

**Authors:** David M. Spranz, Hendrik Bruttel, Sebastian I. Wolf, Felix Zeifang, Michael W. Maier

**Affiliations:** Clinic for Orthopedics and Trauma Surgery, Heidelberg University Hospital, Schlierbacher Landstraße 200a, D-69118 Heidelberg, Germany

**Keywords:** Shoulder arthroplasty, Stemless shoulder arthroplasty, Osteoarthritis, 3D motion analysis, HUX Model

## Abstract

**Background:**

The aim of this study is to compare the functional midterm outcome of stemless shoulder prostheses with standard anatomical stemmed shoulder prostheses and to show that the STEMLESS results are comparable to the STEMMED with respect to active maximum range of shoulder motion (ROM) and Constant score (CS).

**Methods:**

Seventeen patients underwent total shoulder arthroplasty (TSA) in 25 shoulder joints. Stemless TSA was performed in 12 shoulder joints (group STEMLESS), third-generation stemmed TSA in 13 shoulder joints (group STEMMED). Functional results were documented using the CS. 3D-motion-analysis using the Heidelberg upper extremity model (HUX) was conducted to measure active maximum (ROM).

**Results:**

The group STEMLESS achieved a CS of 67.9 (SD 12.0) points and the group STEMMED of 70.2 (SD 5.8 points) without significant difference between the groups (*p* = 0.925). The maximum ROM of the group STEMLESS, ascertained by 3-D-motion-analysis, was in forward flexion 125.5° (SD 17.2°), in extension 49.4° (SD 13.8°), in abduction 126.2° (SD 28.5°) and in external rotation 40.3° (SD 13.9°). The maximum ROM of the group STEMMED, also ascertained by 3-D-motion analysis, was in forward flexion 135.0° (SD 16.8°), in extension 47.2° (SD 11.5°), in abduction 136.3° (SD 24.2°) and in external rotation 40.1° (SD 12.2°). The maximum ROM of the STEMLESS group was lower in forward flexion and abduction, higher in extension and almost identical in external rotation. But there was no significant difference (forward flexion *p* = 0.174, extension *p* = 0.470, abduction *p* = 0.345, external rotation *p* = 0.978).

**Conclusion:**

Both types of shoulder prostheses achieve a similar and good active ROM and similar results in CS.

**Trial registration:**

DRKS00013166, retrospectively registered, 11.10.2017

## Background

As the anatomical TSA has shown quite successful results in reducing pain and improving function when performed in patients with glenohumeral osteoarthritis (OA) and an intact rotator cuff it is the golden standard in surgical treatment. [1, 2]. However, stem-related complications, which include such as bone stock loss, stress shielding, intraoperative and postoperative periprosthetic fractures, mal-positioning of the humeral head component relative to the metaphysis, and in situations of infection difficulty with stem and cement extraction [3–6], have been described in several studies. Therefore, stemless shoulder prostheses, such as the Total Evolution Shoulder System (TESS®; Biomed, France) have been developed to reduce these stem-related complications [3]. Today, different models of stemless TSA are available and increasingly used, but studies about their clinical results are rare [2, 7–11]. The aim of this study is to investigate the clinical midterm outcome of stemless TSA in comparison with a standard anatomical TSA.

## Methods

Seventeen patients (10 female, 7 male) with mean age 72.0 (SD 5.3, range 64-79) years participated in a prospective case series. All patients received a minimum of 3 months of physical therapy (strengthening of the rotator cuff and instruction for self-training) but in the end they suffered from persistent pain.

All participants underwent anatomical TSA. A total of 25 shoulder joints was treated. Two patients were left-handed, 15 patients were right-handed. In 9 cases, only the dominant side was treated, 8 cases were treated bilaterally. Eleven of the participants (5 female, 4 male) with mean age 71.0 (SD 5.4, range 64 - 79) received third-generation stemmed TSA in 13 shoulders (Aequalis Shoulder, Tornier, Lyon, France) with a mean follow-up of 6.3 (SD 2.4, range 3.0 – 11.1) years (group STEMMED). Nine participants (6 female, 5 male) with mean age 74.0 (SD 5.7, range 64-79) received stemless TSA in 12 shoulders (Biomet T.E.S.S., Biomet, Warsaw, USA) with a mean follow-up of 4.3 (SD 1.1, range 2.7 – 6.1) years (group STEMLESS). Inclusion criteria were the diagnosis with primary glenohumeral osteoarthritis with an intact rotator cuff. Exclusion criteria were previous operations at the shoulder and rotator cuff tears. Patients were recruited over a time period of 3.5 months. All patients were operated on by a single surgeon (FZ). By using a deltopectoral approach the subscapularis tendon was detached, the capsular was released and the joint was exposed. In no case a rotator cuff tear was found. A biceps tendon tenodesis was conducted. After the placement of the implants, the subscapularis tendon was reconstructed by using non-absorbable tendon-to-tendon sutures.

To protect the reconstructed subscapularis tendon postoperatively, the operated arm was placed in internal rotation in a shoulder abduction pillow for 4 weeks. The operated shoulder joint was mobilized passively by a physiotherapist with the limitation to 60° of flexion and abduction over a time period of 6 weeks. Patients were requested to support these movements actively. External rotation was strictly prohibited. Free range of motion was allowed 6 weeks postoperatively.

In accordance with the World Medical Association Declaration, the ethics committee of the Heidelberg medical school approved the study protocol. Informed consent was obtained from all individual participants included in the study. All patients were clinically examined to exclude further shoulder pathologies such as shoulder impingement, rotator cuff tear and shoulder instability. Constant Score [12, 13] was obtained for both sides. The Constant Score was used to grade pain (with 0 points indicating severe pain and 15 points indicating no pain), activity of daily living (ADL) (with 0 points indicating no mobility and 20 points indicating full mobility), power (with 0 points indicating 0 kp [0 N] and 25 points indicating 25 kp [110.4 N] and ROM (max. 40 points). Shoulder flexion and abduction were recorded in degrees with a goniometer, whereas external and internal rotation were graded according to landmarks that could be reached by hand. For internal rotation the landmarks were gluteal region, lumbosacral region, 3^rd^ lumbal vertebra, 12th thoracic vertebra and reaching between the scapulas. For external rotation five different positions were tested: Ability to reach over the head, hand on top of the head and hand on the neck both with elbows pointing forward and pointing lateral. The Mann-Whitney-U test was used to search for significant differences.

Afterwards all shoulders were examined via 3D-motion-analysis with an optoelectronic system consisting of 12 infrared cameras (T40-S, Vicon Motion Systems Ltd., Oxford, United Kingdom). All trials were recorded using Vicon Nexus 2 software (Vicon Motion System Ltd., Oxford, United Kingdom). The Heidelberg Upper Extremity Model (HUX) by Rettig et al. [14] was used as a biomechanical model. It used a least-squares method by Gamage and Lasenby [15] to calculate the glenohumeral center of rotation and elbow axis of rotation. Forward flexion/extension and abduction were calculated as projection angles in sagittal and frontal plane respectively. External rotation was calculated as “conjunct rotation” as proposed by Wolf et al. [16]. For reference a coordinate system for the thorax was defined by incisura jugularis, processus xiphoideus, processus spinosus of 7th cervical and 10th thoracic vertebra [14] essentially following the recommendations of the International Society of Biomechanics, which propose using the 8^th^ thoracic vertebra [17]. All angles were calculated by using customized Java software. Subjects were asked to move their arm back and forth between maximum forward flexion and maximum extension with elbow fully extended to assess ROM in sagittal plane. Movements from neutral position to maximum abduction were used to assess ROM in frontal plane. Adduction was not accounted for, as visibility of thorax markers would have been restricted by the upper arm. Rotation was tested by moving arm back and forth between maximum external rotation and hand-to-belly position with elbow flexed by 90°. As it did not resemble maximum internal rotation only external rotation was evaluated. Between one to four repetitions could be evaluated and the maximum achieved position was used. Minimum and maximum values were calculated using Matlab R2015a (The MathWorks Inc., Natick, USA). The Shapiro-Wilk test was used to test for normal distribution. The Levene’s test was used to assess equality of variance. The Mann-Whitney-U test was used for non-normally distributed data. Normally distributed data were analyzed using Student’s t-test. *P*-values < 0.05 were considered significant. All statistical analysis was performed in SPSS 23 (International Business Machines Corporation, Armonk, USA).

## Results

**Fig. 1:**
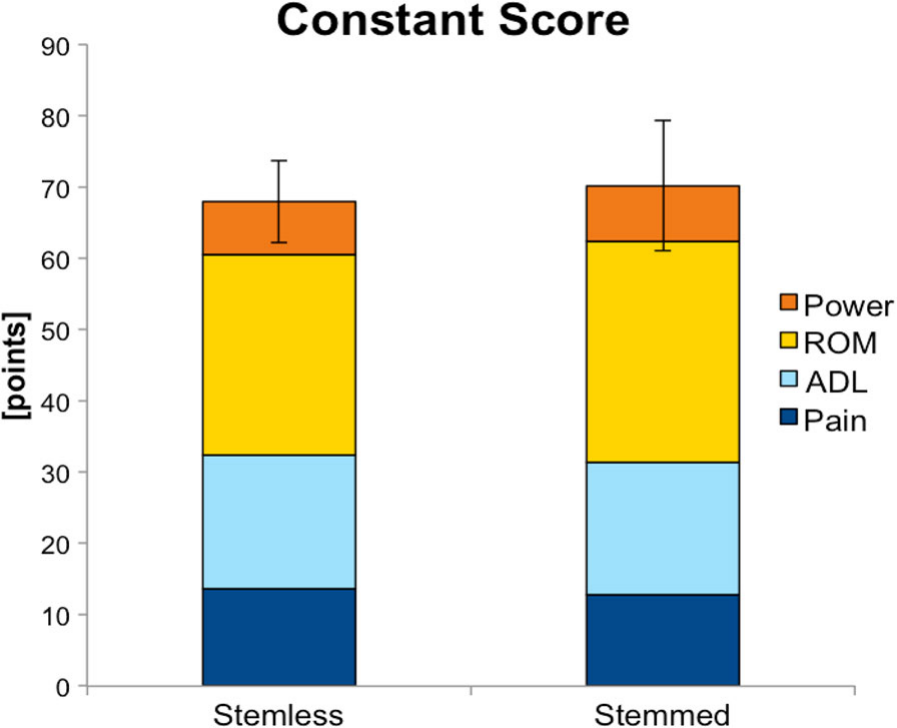
Postoperative Constant Score of the group STEMLESS and the group STEMMED

**Fig. 2:**
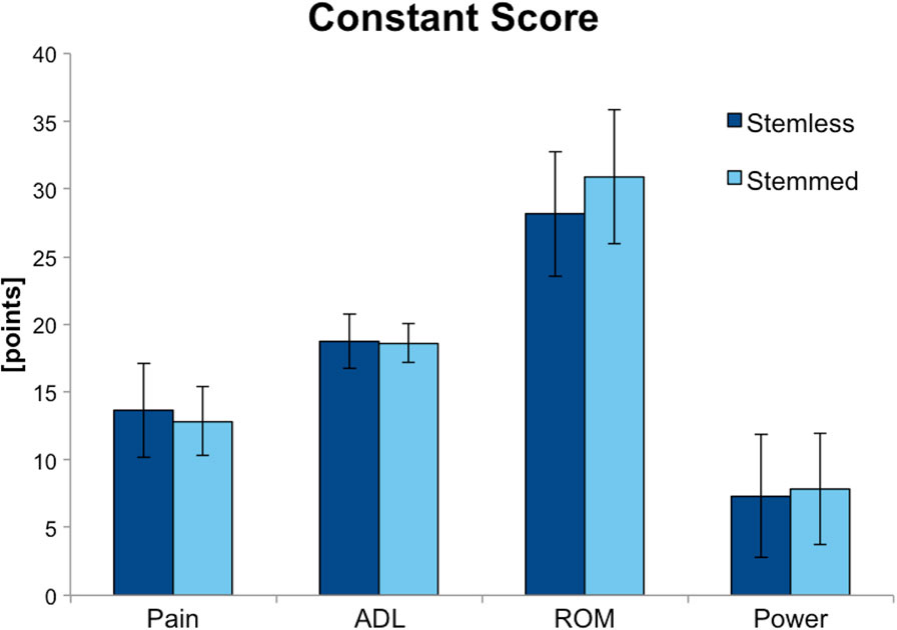
Postoperative Constant Score categories pain, ADL, ROM, and power of the group STEMLESS and the group STEMMED

The group STEMLESS achieved a Constant Score of 67.9 (SD 12.0) points and the group STEMMED of 70.2 (SD 5.8) points without significant difference between the groups (*p* = 0.925) (see Fig. 1). The categories pain, ADL, ROM and power did not show any significant differences either (see Fig. 2 and Table 1).

**Table 1:** Postoperative Constant Score of the group STEMLESS and the group STEMMED

	STEMLESS	STEMMED	*p*-value
Pain (/15)	13.7 ± 3.5	12.8 ± 2.5	0.167
ADL (/20)	18.8 ± 2.0	18.6 ± 1.4	0.535
ROM (/40)	28.2 ± 4.6	30.9 ± 4.9	0.283
Power (/25)	7.3 ± 4.5	7.8 ± 4.1	0.543
Total (/100)	67.9 ± 12.0	70.2 ± 5.8	0.925

**Fig. 3:**
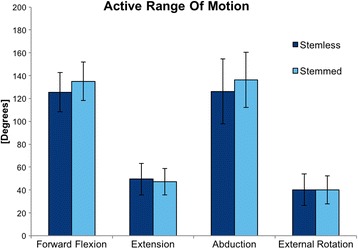
Postoperative maximum active ROM of the group STEMLESS and the group STEMMED in forward flexion, extension, abduction, and external rotation

The maximum ROM of the group STEMLESS, ascertained by 3-D-motion-analysis, was in forward flexion 125.5° (SD 17.2°), in extension 49.4° (SD 13.8°), in abduction 126.2° (SD 28.5°) and in external rotation 40.3° (SD 13.9°) (see Fig. 3 and Table 2).

**Table 2:** Postoperative maximum active ROM of the group STEMLESS and the group STEMMED in forward flexion, extension, abduction and external rotation

	STEMLESS	STEMMED	*p*
Forward Flexion	125.5 ± 17.2	135.0 ± 16.8	0.174
Extension	49.4 ± 13.8	47.2 ± 11.5	0.470*
Abduction	126.2 ± 28.5	136.3 ± 24.2	0.345
External Rotation	40.3 ± 13.9	40.1 ± 12.2	0.978

*Mann-Whitney-U test was used to assess Extension as Shapiro-Wilk test indicated that data was not normally distributed

The maximum ROM of the group STEMMED, also ascertained by 3-D-motion analysis, was in forward flexion 135.0° (SD 16.8°), in extension 47.2° (SD 11.5°), in abduction 136.3° (SD 24.2°) and in external rotation 40.1° (SD 12.2°) (see Fig. 3 and Table 2).

The maximum ROM of the group STEMLESS compared with the group STEMMED was lower in forward flexion and abduction, higher in extension and almost identical in external rotation. But there was no significant difference (forward flexion *p* = 0.174, extension *p* = 0.470, abduction *p* = 0.345, external rotation *p* = 0.978) (see Table 2).

## Discussion

It has been shown that the prosthetic anatomic stemmed TSA produces quite successful results in reducing pain and improving function when performed in patients with OA and an intact rotator cuff in the long-term follow up and is therefore the golden standard in the surgical treatment [18]. The Aequalis TSA is an unconstrained third-generation prosthesis with variable medial and posterior offset which replicates the complex shape of the proximal humerus. This type of stemmed TSA has shown excellent results in long-term follow up studies [18, 19].

The restoration of the individual anatomy of the glenohumeral joint with superior reconstruction of the humeral head geometry is an essential factor for postoperative functional results [9]. Possibilities to adjust the position of the head component are limited in stemmed TSA. In case of the stemmed Aequalis prosthesis the inclination can only be modified in steps and the offset of the center of rotation can only be set along a simple eccentric track. Stemless TSA has been performed since 2004 in Europe [11]. The stemless system enables the individual anatomic reconstruction of the center of rotation of the humeral head without any external restraints, e.g. the shaft axis [11]. Moreover stemless TSA might reduce the risk of perioperative bleeding, and the operative time seems to be lower [3, 7, 9, 20]. Furthermore, in case of revision, the integrity of the humeral shaft and neck more easily allows further implants [20].

The results provided by stemless TSA must be compared with the very good results achieved by using the existing anatomic stemmed TSA concept.

In this study, we have compared the clinical results of patients with primary OA of the shoulder who had been surgically treated with either a standard stemmed TSA or an anatomical stemless TSA.

The aim of this study has been to investigate the clinical midterm outcome of stemless TSA in comparison with a standard anatomical TSA related to the CS and the active ROM.

In our study we have used the HUX-Model, described previously by Rettig et al. [14] and applied in some studies [21–23] to evaluate the maximum range of motion. It promises high objectivity and high intra- and interrater reliability.

Only few studies have reported the clinical results of stemless shoulder arthroplasty in the short-term follow-up. All these studies have used a goniometer.

Schoch et al. [24] once reported early results of 96 stemless TSA with primary OA with a mean follow-up of 13.2 month (± 3.5). The patients achieved significant improvement of the absolute Constant Score from 44 points preoperatively to 66 points at follow up. ROM improved significantly with forward flexion of 145°, abduction of 105° and external rotation of 41° [24]. Huguet et al. [9] reported results of stemless TSA with a mean follow-up of 45 months in 63 patients. The Constant Score increased significantly from 29.6 points preoperatively to 75 points postoperatively. Active forward flexion improved to 145° and external rotation improved to 40°. The author concluded that the early results of stemless TSA improved functional results similar to those of third-generation and fourth-generation stemmed implants [9].

Habermeyer et al. [11] reported results of stemless TSA in midterm follow-up with a mean follow up of 72.9 months. The constant score improved significantly from 38.1 to 75.3 points. Active range of motion, ascertained by using a goniometer, improved significantly for forward flexion (from 114° to 141°), abduction (from 74° to 130°) and external rotation (from 25° to 44°). The author concluded that the functional results of stemless TSA were comparable to third and fourth generation of standard stemmed TSA in midterm follow-up [11].

In accordance with this study we have detected no significant difference in the maximum ROM or in the Constant Score in the midterm follow-up between stemless and stemmed shoulder arthroplasty.

Thus we can compress that the stemless shoulder prosthesis provides very good clinical results in patients with primary OA without the need of a humeral stem.

One limitation of our study was the short duration of our follow-up. Our assessment with a mean follow-up of 4.3 years in the TESS group was too short to detect possible differences in the survivorship of stemless TSA. Further investigations would be necessary to determine the long-term performance of this kind of prosthesis. Another limitation was that some patients included in this study were treated and examined on both sides while others only were treated unilaterally. Although the results can be regarded as the results of independent surgical treatments, patient factors such as postoperative training motivation and compliance, pain perception, handedness and others possibly have an impact on the overall outcome that may influence both shoulders and thus violate the assumption of independence of statistical testing.

But we would like to record the fact that, to our knowledge, there is currently no other study with a longer follow-up. Moreover, using the HUX-Model promises high objectivity and high intra- and interrater reliability.

## Conclusion

Both types of shoulder prostheses achieve similar results for active ROM and CS in the midterm follow-up.

## Abbreviations

ADL: Activity of daily living
CS: Constant score
HUX: Heidelberg Upper Extremity Model
OA: Glenohumeral osteoarthritis
ROM: Range of shoulder motion
TSA: Total shoulder arthroplasty
